# Tactile and Somatic Hallucinations in a Muslim Population of Psychotic Patients

**DOI:** 10.3389/fpsyt.2021.728397

**Published:** 2021-10-29

**Authors:** Anastasia Lim, Jan Dirk Blom

**Affiliations:** ^1^Outpatient Clinic for Uncommon Psychiatric Syndromes, Parnassia Psychiatric Institute, The Hague, Netherlands; ^2^Faculty of Social and Behavioural Sciences, Leiden University, Leiden, Netherlands; ^3^Department of Psychiatry, University of Groningen, Groningen, Netherlands

**Keywords:** bodily hallucination, entity experience, multimodal hallucination, schizophrenia spectrum disorder, transcultural psychiatry

## Abstract

**Background:** Tactile and somatic hallucinations are distressing phenomena that have hardly been researched. The few studies that have been published focus on their occurrence in neurodegenerative disorders and substance use, and, surprisingly, not on schizophrenia spectrum disorders.

**Objective:** To fill this gap in our knowledge, we sought to explore the phenomenological characteristics of tactile and somatic hallucinations in a group of psychotic Muslim patients. Since many Muslims attribute such experiences to jinn (invisible spirits) and jinn are often perceived in multiple sensory modalities, we not only charted the involvement of the tactile and somatic modalities but also their interrelatedness with hallucinations in other sensory modalities.

**Methods:** We performed a cross-sectional study using a semi-structured interview and dedicated questionnaire.

**Results:** Of the 42 Muslim inpatients mostly diagnosed with a schizophrenia spectrum disorder, 62% reported to suffer from tactile and/or somatic hallucinations. Their phenomenological characteristics varied, with 96% being multimodal in nature and 38% taking the form of full-blown entity/jinn encounters. In comparison to other entity experiences, the involvement of the tactile and somatic modalities was exceptionally high, as was the level of ensuing distress. Regarding the order of recruitment of the various sensory modalities, we suggest the involvement of an underlying stochastic process.

**Conclusion:** Muslim patients with severe psychosis can have tactile and somatic hallucinations, of which a large number are multimodal or full-blown entity/jinn encounters, which are almost invariably appreciated as harrowing. On the basis of our findings we make recommendations for further research and clinical practice.

## Introduction

Tactile and somatic hallucinations are classified as bodily hallucinations (1, 2), i.e., hallucinations of the “proximity senses” (Table 1), as opposed to hallucinations of smell, sight and hearing, which have bearing on the “distance senses.” These phenomena have been overly neglected in literature and research, while both modalities are clinically relevant. Touch, as one of the main senses, is such a common and continuous presence in our everyday lives that we mostly take it for granted. Provided that we are comfortable and at ease, we hardly notice the pressure shoes or clothing exert on our feet and skin, or the weight of glasses on our nose and ears, or even the accidental touch of fellow travelers on a busy morning train. Dating back at least to Aristotle, touch has, moreover, been ranked as the lowest and most ordinary of the senses, providing either pain or pleasure but nothing that transcends these “bestial” feelings. Together with our relative lack of awareness of information generated by the body surface, that may well be the reason why this sensory modality has received less scientific attention than others. One might say, though, that touch has thus been gravely neglected. Touch serves crucial communicative and protective functions. We need it to establish the body's boundaries, to determine what is inside and outside of us, and to assess whether our contacts and surroundings are safe and healthy or whether perhaps a painful and potentially fatal transgression is imminent. In addition, touch allows us to integrate the input from the other sense organs and to thus create a coherent whole that we experience as the body or “self.” Therefore, losing our sense of touch—as in acenesthesia, for instance—means nothing less than losing our sense of body ownership and, with that, losing our sense of identity (3). Thus, touch does not only tell us *where* we are with respect to our immediate surroundings but also *who* we are (4). Last but by no means least, touch is a compelling indicator of what is real and what is not real in the outside world. Despised and neglected as it may be as a sensory modality, we trust it more than any of the other senses when we seek to verify perceptual information. Seeing or hearing an object is one thing, touching it means that it is actually there. If touch thus has the dual, almost mystical connotation of “the sense that makes us human” and “the sense that tells the truth,” what then are the implications of being touched by unseen forces, to experience touch where no external source or cause can be discerned? This is what the present paper is about.

**Table 1:** Classification of bodily hallucinations and concomitant sensory modalities [from (2)].

**Sensory modality**	**Type of hallucination**
(1). Exteroceptive modality	Tactile (haptic) hallucination
(2). Interoceptive modality	Somatic hallucination, visceral hallucination
(3). Proprioceptive modality	Proprioceptive hallucination
(4). Kinesthetic modality	Kinesthetic hallucination
(5). Vestibular modality	Vestibular hallucination
(6). Coenesthetic modality	Coenesthetic hallucination
(7). Pain modality	Hallucinated pain, central pain
(8). Sexual modality	Sexual hallucination
(9). Temperature modality	Thermal (thermic) hallucination

### Tactile Hallucinations

Tactile hallucinations are also known as haptic hallucinations and tactile phantasmata (5). They are defined as sensations of touch in the absence of a corresponding stimulus from the outside world and characterized by apparent touch to the skin, including, sometimes, the underlying tissues. They can mimic all kinds of bodily sensations normally registered by the skin, such as pin pricks, the sensation of fluids or the wind upon one's skin, a hand on one's shoulder, or a blow to the face. Pathophysiologically, they are associated with activity in sensory cortical areas subserving the skin and subcutaneous tissues. Over the past 15 years, case reports and modest case series have reported on tactile hallucinations in varying conditions, including neurodegenerative disorders (6), illicit drug use (7), and medication use (8–11). Rare as they may seem in psychiatric and neurological conditions, in the general population they were found to be the second-most prevalent type of hallucination. Thus, the NEMESIS II trial, a large cohort study carried out in the Netherlands from 2007 through 2015, mentioned 0.31% as the yearly incidence for tactile hallucinations (the first place being occupied by visual hallucinations, with a slightly higher rate of 0.33%; 12). The study also emphasized the clinical relevance of tactile hallucinations through associations with poorer quality of mental health and risk factors for psychosis.

### Somatic Hallucinations

Somatic hallucinations are defined as bodily sensations experienced *inside* the body, in the absence of an objectifiable source or cause. At times it is difficult to differentiate these phenomena from unexplained medical complaints, in the sense that local pathology in the thorax or the abdomen, for instance, can be either overlooked or simply remain undiagnosed despite current state-of-the-art procedures. Examples of somatic hallucinations are the sensation of having a lump in the throat that is not there, of animals crawling through the body, of a plant scraping its pointed leaves against the inside of one's skull, or bones apparently increasing or decreasing in size. The literature on somatic hallucinations is even less prolific than that on tactile hallucinations (12, 13). Case descriptions and small case series chiefly report on these phenomena in the context of brain lesions (12), temporal-lobe epilepsy (14), and intoxications (7, 8). In schizophrenia spectrum disorders they are considered to be rare. When they have been documented at all, it is mostly in combination with other psychotic symptoms [e.g., (15–17)]. Pathophysiologically, somatic hallucinations are mainly attributed to activity in the secondary somatosensory cortex, posterior parietal cortex (15, 18), and limbic regions (19).

In general, cultural patterns and expectations can affect hallucinations in those who experience them (20) and therefore should be taken into account. The gap between the medical approach of hallucinations, often the first tactic taken on by Western health professionals, and the patient's own cultural point of view, is a common theme in psychiatric settings. The present study examines tactile and somatic hallucinations in a rather specific population, namely in Muslim patients diagnosed with a psychotic disorder who are liable to attribute their hallucinations to jinn, i.e., invisible beings created by Allah. Among Muslims in general, it is not uncommon to attribute setbacks in life—whether they are of a medical, psychological, social, religious, or even financial nature—to jinn (21). As a consequence, many Muslims consider the existence of jinn a given. To perceive them, on the other hand, is generally seen as an indication that the natural order of things is broken (22). In the context of what biomedicine considers to be psychosis, these entities are often experienced in more than one sensory modality at the same time.

Since it is unknown to what extent the tactile and somatic modalities are involved in such instances, and since these hallucinations have hardly been researched in people diagnosed with schizophrenia spectrum disorders or in patients with a Muslim background, below we describe their phenomenological characteristics and assess their relevance for clinical practice and further scientific research. Of note here is that we will approach these sensations in this article from a biomedical perspective only, although taking into account the knowledge and experience of our previous research on Muslim explanatory models of disease. We have no intention to pass an opinion on the ontological status of said entities from a religious or cultural point of view.

## Methods

We performed a cross-sectional phenomenological study among Muslim patients under care at Parnassia Psychiatric Institute, The Hague, the Netherlands, in the period of 2008–2014. Inclusion criteria were age ≥18 years, a Muslim background, the self-reported presence of hallucinations, the attribution of these hallucinations to jinn or related culturoreligious entities, and a sufficient mastery of Dutch or English. All participants were (or had recently been) admitted to our hospital.

All participants were interviewed with the aid of the Hallucination Attribution List (HAL), an 80-item, purpose-built, semi-structured questionnaire that systematically assesses the presence of (lifetime and present-state) hallucinations, the sensory modalities involved, and the attribution of these phenomena—in conformity with Muslim culturoreligious practices—to jinn, voodoo, and/or the evil eye (Supplementary Material). When participants reported hallucinations in the tactile or somatic modalities, the phenomenological characteristics of their sensations were explored, as well as their interrelatedness with other types of hallucination—if present—in the auditory, visual, olfactory, and gustatory modalities. The study was approved by the Scientific Committee of the Parnassia Academy and considered exempt from independent ethics review, and was carried out in accordance with the revised conditions of the Declaration of Helsinki.

## Results

In our convenience sample of 42 Muslim patients admitted to our inpatient center in an acute clinical state, 62% (*n* = 26) reported bodily, i.e., tactile and/or somatic, hallucinations. Of these, 69% (*n* = 18) were men. Most participants reporting hallucinations (*n* = 21, 81%) were diagnosed with a schizophrenia spectrum disorder following the DSM-IV criteria. In nine cases (35%) a second diagnosis was made (see Table 2 for details). The participants' mean age was 31 years (range 22–52 years). Of the 26 patients with tactile or somatic hallucinations only one had unimodal tactile hallucinations; he was diagnosed with bipolar disorder. None made mention of unimodal somatic hallucinations.

**Table 2:** Diagnoses of the participants experiencing tactile and/or somatic hallucinations (*N* = 26).

**Primary diagnosis**	***n***	**Secondary diagnosis**	***n***
Schizophrenia spectrum disorder	21	Major depressive disorder	2
		Cannabis use disorder	3
		Posttraumatic stress disorder	1
		Substance use disorder (multidrug use)	2
		Social anxiety disorder	1
Bipolar disorder	1	Sensed presence and incubus phenomenon	
Posttraumatic stress disorder	1	Incubus phenomenon	
Major depressive disorder with psychotic symptoms	1	Incubus phenomenon, epilepsy, and personality disorder	
Major depressive disorder	1	Panic disorder with agoraphobia	
Major depressive disorder	1	Incubus phenomenon	

In the total group of 42 patients, 23 (55%) experienced tactile hallucinations. Fourteen patients experienced somatic hallucinations (33%).

Of the 26 patients with tactile/somatic hallucinations, all but one experienced multimodal hallucinations, in essence in 96% (*n* = 25 of 26 patients) in this subgroup focused on. In the total group of patients who were interviewed, 60% (*n* = 25 of the total group of 42 patients) had experienced multimodal hallucinations.

There were 23 patients who experienced tactile hallucinations (either unimodal or multimodal) (88%) and 14 who experienced somatic hallucinations (54%). In the group of 26 patients, there were 12 who experienced tactile and somatic hallucinations simultaneously or serially (Tables 3A,B)

**Table 3A:** Interrelatedness of the sensory modalities in the patients having tactile hallucinations (*n* = 23).

		**Tactile**	
**Number of sensory modalities**	**Serial congruent *n*=**	**Serial congruent and simultaneous congruent *n*=**	**Simultaneous congruent *n*=**
2	2		1
3	1	3	1
4	3	1	
5	1		6
6	1	2	1
	8	6	9

**Table 3B:** Interrelatedness of the sensory modalities in the patients having somatic hallucinations (*n* = 14).

		**Somatic**	
**Number of sensory modalities**	**Serial congruent *n*=**	**Serial congruent and simultaneous congruent *n*=**	**Simultaneous congruent *n*=**
2			
3	1	1	
4	3		
5	1		4
6	1	2	1
	6	3	5

In eight of the 23 cases (35%) having tactile hallucinations, these consisted of *serial, congruent* multimodal hallucinations, with the involvement of at least two sensory modalities, meaning that the hallucinations were attributed to the same source (e.g., jinn) but experienced at different moments in time. In 39% they were described as *simultaneous, congruent* hallucinations, reflecting that they were attributed to the same source and experienced at the same moment in time, in conformity with the multimodal nature of entity experiences. In the case of somatic hallucinations, 43% were serial and congruent, and 36% simultaneous and congruent. Tables 3A,B detail these interrelations. In 91% of the participants having tactile hallucinations, auditory hallucinations were also present, while 87% experienced these in combination with visual hallucinations. The co-occurrence of tactile hallucinations with olfactory and gustatory hallucinations was reported by 57 and 30%, respectively. We found comparable rates for somatic hallucinations; these coincided with auditory, visual, and tactile hallucinations in 86%, with olfactory hallucinations in 79%, and with gustatory hallucinations in 43%.

### Phenomenology

The way in which the participants experienced their tactile and somatic hallucinations showed considerable interindividual variation. Table 4 sums up the phenomenological characteristics. Ten participants (38%) only recounted of a single hallucinated sensation, whereas the other 16 (62%) described two or more sensations (e.g., the feeling of being stabbed *and* suffering electrical shocks). In Table 4 the category ‘different sensations’ comprises a motley collection of tactile phenomena, including being tapped on the back, being groped and/or squeezed, feeling nails being hammered into the head, being kicked, being bitten, and being grabbed by the throat. One participant also described the sensation of someone stepping on her blanket while she was lying awake in bed. We did not find such a broad range of sensations in the group reporting somatic hallucinations. Here, they were limited to the feeling of something entering the body, something moving inside the body, and sexual sensations (including orgasmic experiences). Regarding the sexual sensations, one participant reported being stroked and sexually abused while he was awake, while another participant reported experiencing multiple orgasms.

**Table 4:** Phenomenological characteristics of the tactile and somatic hallucinations reported.

	**Tactile hallucinations**	**Somatic hallucinations**
**Phenomenological characteristics**		
Feeling of being pushed	4	
Feeling of being hit	4	
Feeling of being stabbed	7	
Feeling of wind	7	
Feeling of water on the skin	2	
Burning sensation	4	
Electrical shock	5	
Different	13	
Feeling of something moving inside or into the body		9
Feeling of sexual arousal and/or stimulation		4

The patient with unimodal tactile hallucinations was in doubt whether his hallucinations were caused by jinn, with a conviction of 50%. Most patients (*n* = 15, 58%) were fully convinced (100%) that their experiences were caused by a jinn, while in the remaining 10 patients (38%) this conviction was lower than 90%, with the majority (*n* = 6, 23%) rating the degree of conviction at 30–60% and one participant indicating that he could not tell whether this should perhaps be 0% even though he insisted on having (had) personal experience with jinn. The participants expressing doubt about the involvement of jinn mentioned alternative causes for their tactile and somatic sensations such as Allah, God, Iblis, a ghost, an actual person, their own brain, and “disease”.

Phenomenologically, the hallucinations were quite diverse. Almost 40% reported simultaneous, congruent multimodal hallucinations, thus constituting full-blown entity experiences. One of our participants indeed described how he saw a female jinn walking toward him, felt her entering his body, and then giving birth to a baby inside him. When the jinn subsequently left his body, he kept feeling the baby moving inside his abdomen and even heard it crying from time to time. Another participant recounted how, for years, he recurrently saw and felt a small jinn nestle itself on his face when he was lying on his back, attaching itself so firmly that he could hardly breathe. The sensation was so frightening that, when he was scheduled to have an MRI scan he requested a junior physician to accompany him inside the scanner room so that she could help him when he would signal its presence. Yet another participant had the gruesome experience of feeling a jinn crawling up against his buttocks, positioning itself anus-to-anus, and squirting feces into his rectum. As these examples illustrate, the hallucinations our participants were experiencing were not only harrowing but also phenomenologically quite diverse. And yet, they did not even cover the entire spectrum of bodily hallucinations (Table 1).

## Discussion

In our convenience sample of 42 psychotic inpatients with a Muslim background, a relatively high rate (62%) acknowledged to have tactile and somatic hallucinations, the far majority of which were denoted as unpleasant, threatening and/or painful, and all of them being attributed to jinn (albeit with varying degrees of certainty). Our patient group mainly reported tactile, somatic, and sexual sensations. Although not actively probed, proprioceptive and kinesthetic hallucinations, for example, went unreported. Nonetheless, the severity and impact of the tactile and somatic hallucinations imparted to us were impressive. Moriyama et al. (23) already pointed out the clinical relevance of tactile hallucinations for the general population in terms of an increased risk of developing psychosis. When tactile and somatic hallucinations occur as described above, it is quite imaginable that patients appreciate such a multidimensional breach of the boundaries of their bodies as an extreme threat to “the self” and thus as most terrifying. Thus the normal, “primary” belief in jinn may be replaced by abnormal, “secondary” beliefs (in the sense of overvalued ideas and/or delusions) when jinn are experienced as entities in the perceptual domain. The relatively high prevalence of bodily hallucinations in our group might be explained by the high prevalence of hallucinations in the auditory and/or visual modalities, which may be a necessary condition to trigger bodily hallucinations *via* a stochastic process (to be explained below). In clinical practice, Western health professionals may be inclined to treat these phenomena as hallucinations and delusions in the context of psychotic disorder. In the literature, evidence for the effects of antipsychotics on tactile and somatic hallucinations is only presented in case reports, with a favorable role for olanzapine (24, 25). However, there is a more extensive body of literature that stresses the importance—in cases such as these—of a culture-sensitive approach which may include religious counseling and/or culturally adapted cognitive-behavioral therapy (16, 26, 27).

### Religious Perspective

The examples above suffice to illustrate the impact of tactile and somatic hallucinations on the population we studied. We did not formally quantify the levels of experienced distress, but the far majority of our participants were so terrified that they had taken refuge to religiously and culturally sanctioned treatments—often for years or even decades on end, spending considerable amounts of money in the process—before seeking the advice of biomedical practitioners. The belief in jinn is an integral part of Islamic faith and of the body of folk beliefs prevalent in Muslim societies (28). They are said to live among us and to go about their business much the same as humans. Reportedly being created out of smokeless fire, they are deemed invisible to the human eye. However, according to religious authorities they can make themselves visible if they so wish. According to others, they will merely take the shape of an animal or other creature without revealing their true identity. Either way, for a human to perceive jinn, if only by means of the distant senses, is considered utterly alarming and a clear sign that the natural order of things has been breached. Even though jinn can allegedly be as virtuous or immoral as we humans can be, with numerous gradations in between, customarily a distinction is made between good and bad jinn. Among the jinn appraised as “bad,” those who touch or transgress the boundaries of the human body are generally considered the most dangerous. According to Islamic folk belief, the body is like a fortress that has doors and windows but also invisible fissures in the skin through which jinn can slip inside (22). People who keep their bodies, hearts, and souls pure are believed to be relatively immune to jinn thanks to the light that shines within them, but those who do not may find their bodies' apertures growing wider, thus rendering themselves more vulnerable to jinn. As Drieskens (22) summarizes:

*The cause of weakness toward djinns is always a combination of factors. The susceptibility to djinns increases when the orifices grow wider and the protecting light diminishes. Various circumstances can cause this, but fear, confusion, doubt and pollution are often involved* [(22), p. 168].

To Muslims, bodily hallucinations may therefore not only indicate the dangerously close proximity of jinn—or even suggest a state of possession—they also implicitly signal one's religious, moral, and/or bodily inferiority, which may be yet another source of anxiety, and, along with that, embarrassment. This, in turn, may prompt people to keep their experiences to themselves and to suffer in silence, even for long periods of time. When they finally decide to seek help, they will tend to turn to a professional knowledgeable of these entities, such as an imam, traditional healer, or Muslim religious counselor—which for them is the logical thing to do. After all, why would one expose oneself to a Western-style health professional who cannot be expected to understand matters such as these and who is, moreover, powerless in the face of such actual, living entities? And, on top of that, why would one risk yet another type of stigma, the likelihood of being branded a psychiatric patient (26)?

### Developmental Perspective

One can also explain the impact of bodily hallucinations from the vantage point of our ontogeny. The proximity senses are the first means by which the embryo registers signals from its environment, with the distant senses developing at a later stage of gestation. Ontogenetically, the skin, the organ of touch, thus is the oldest of our sense organs. With a mean surface area of 1.7 m^2^ in adults, it is also the largest. Located at the crucial interface of the body and the surrounding world, it is connected with the spinal cord and brain by a quarter million tactile afferent fibers (29). Together with the skin's tactile receptors, these anatomical structures enable us to discern an almost infinite range of tactile sensations. Throughout our lives, the skin also forms the flimsy barrier between the inside and the outside world, enveloping and protecting us. Without it, we would soon succumb to the multitude of unfiltered stimuli that assail us (which would then directly affect the underlying tissues) and lose our ability to integrate information coming from the other senses. Ironically, for such an important sensory modality, touch is the first but also—as we saw—the most neglected sense (30). And yet, it has furnished us longer than any of the other senses with multiple perceptions that are all of vital importance to our functioning and survival. Their range is reflected in the equally wide range of hallucinatory phenomena that we are capable of experiencing. That so many people report these phenomena (23) *and* tend to typify them as impactful may then well be due to the fundamental role touch plays from an early embryonic stage onwards.

### Comparison With Other Entity Experiences

Jinn are not the only entities that have been the subject of analyses of multimodal hallucinations. Recently, studies have been published on tiny hallucinated creatures (lilliputian hallucinations) (31), entities perceived in the context of dimethyltryptamine (DMT) use (32), and God experiences (in non-drug-using individuals and in those using four types of hallucinogen) (33). In all four studies multimodality played a substantial role. However, only 9–43% of the participants in these studies reported the involvement of the tactile and somatic modalities, where this is 62% in the present study. Since the other four types of entity experience were much more often appraised as agreeable, we may ask ourselves whether the high levels of distress associated with jinn encounters are connected with (i) the catastrophic beliefs of our patients concerning jinn in general, (ii) the threatening way in which these entities frequently appear to behave, (iii) the psychiatric disorders in the context of which they were encountered, (iv) the duration of the experience (which in our study often amounted to years), or (v) the overrepresentation of tactile and somatic components in the experiences recounted. Likely, the answer lies in a combination of these factors, if only because the episodes during which the participants in the four previously published studies had had their entity encounters often were much shorter, while fewer participants were diagnosed with a psychiatric disorder. In a chicken-and-egg type of unresolved issue, the tendency of our study population to react with horror to the entities they perceived may also have increased the likelihood that they would develop a psychotic disorder. Since a negative appraisal of hallucinations is known to be an important predictor of schizophrenia spectrum disorders, at least in the case of auditory hallucinations (34), this might well be a factor to reckon with. If we also call to mind Moriyama et al.'s finding (23) that tactile hallucinations in the general population are linked to poorer mental health and an increased risk of psychosis, we may conclude that multiple factors are at play that together increase the likelihood that jinn are experienced as distressing entities *and* that a biomedical diagnosis of schizophrenia spectrum disorder is probable.

### Stochastic Process

The order in which the various sensory modalities are engaged in multimodal hallucinations is a topic of emerging interest (16, 35). In schizophrenia spectrum disorders, for example, auditory hallucinations often appear to precede hallucinations in the visual and tactile modalities (16). The reasons for this are unclear, although Hoffman's (36) social deafferentiation hypothesis stresses the vital importance of language—in a social and evolutionary sense—and accordingly proposes that hallucinated speech is the first symptom to arise in the context of social isolation, which often precedes and/or accompanies psychosis. By contrast, hallucinations triggered by sleep deprivation almost invariably start out in the visual modality, sometimes followed by auditory and tactile sensations, and finally by cognitive failure in terms of delusions (37). Thus, in severe sleep deprivation, the recruitment of brain areas responsible for the mediation of hallucinations appears to proceed from occipital toward prefrontal areas. We found that the tactile hallucinations our psychotic Muslim patients reported seem to chiefly co-occur with auditory and visual hallucinations, which suggests that they only appear when at least one other sensory modality—and often two—are already involved. We observed a somewhat similar pattern for the somatic hallucinations, in that these only seem to appear when at least two other sensory modalities are involved, with 64% of the participants reporting hallucinations in as many as four or five other modalities. Limited by the cross-sectional nature of our study, we cannot draw any firm conclusions about the order of recruitment of these sensory modalities, but Tables 3A,B do hint at a stochastic process where other sensory modalities need to have been engaged first in order for tactile and especially somatic hallucinations to appear.

### Limitations

The group of patients participating in our study was relatively small and, moreover, comprised a subgroup at the severe end of the psychosis spectrum. The generalizability of our findings—even to other Muslim patients with psychosis—is therefore questionable.

For the purpose of charting the phenomenological characteristics of bodily hallucinations we did not consider PANSS scores and medication histories as strictly necessary at this point, and therefore refrained from including these methods in our study design. In the future, these sources of information might have the potential of adding valuable information.

Also, since the structure of the questionnaire we used did not allow us to chart the full spectrum of bodily hallucinations, proprioceptive and kinesthetic hallucinations, for example, remain to be explored. Also, the cross-sectional nature of our study prevented us from establishing the order in which the various sensory modalities were recruited. What is more, the lack of a control group—and the scarcity of published studies on bodily hallucinations in other populations—prevents us from drawing any firm conclusions regarding the question whether these phenomena are overrepresented in Muslim patients. Finally, although the majority of our participants reported high levels of distress, we did not formally quantify its extent. As a consequence, we were unable to relate the intensity of distress to the types or number of sensory modalities involved. This work provides a first step of describing the phenomenology of tactile and somatic hallucinations. These descriptions can be very useful in the further development of culturally appropriate assessments and treatments. Future research might benefit from (i) broadening the scope of this research by including all varieties of bodily hallucination (as well as their relation with hallucinations of the distance senses), (ii) a prospective design that would allow the order in which the various sensory modalities are recruited to be determined, (iii) a quantification of the extent of distress in relation to the sensory modalities involved *and* to entity experiences of the jinn type, and (iv) functional imaging to shed further light on the brain networks implicated in the various types of hallucination.

## Conclusions

Bodily hallucinations have long been neglected by clinicians and researchers. This may have to do with the fact that touch is traditionally ranked as one of the “lower” senses but also with the prominence of auditory and visual hallucinations in psychiatric and neurological conditions, ranging from psychotic disorder to Parkinson's disease and Charles Bonnet syndrome. We have shown that at least in a subgroup of psychotic patients tactile and somatic hallucinations deserve our full clinical attention. Of the 42 Muslim patients clinically admitted with severe psychosis, 62% reported to suffer from such hallucinations, with several describing sexual hallucinations. In 96%, the hallucinations were of a multimodal nature, of which almost 40% took the form of full-blown entity experiences. The involvement of the tactile and somatic modalities was exceptionally high in our group, as was the level of concomitant distress, which may be due to the strong belief most claimed to have in the objective existence of jinn. This, along with the anxiety generally associated with these beings, may have helped to shape their hallucinations, explaining the prominent role of bodily sensations. Also or alternatively, the relatively long duration of illness in our group may have allowed for the recruitment of the tactile and somatic modalities, which we believe become involved in a relatively late stage compared to other sensory modalities when people experience hallucinations in the context of schizophrenia spectrum disorders. Although the cross-sectional nature of our study does not allow for firm conclusions regarding this issue, we found indirect evidence for a stochastic process underlying this recruitment process. The high levels of distress our participants reported indicate that both bodily hallucinations and jinn (encounter) experiences deserve closer attention in clinical practice, as do similar phenomena in patients with analogous culturo-religious backgrounds.

## Data Availability Statement

The original contributions presented in the study are included in the article/supplementary material, further inquiries can be directed to the corresponding author.

## Ethics Statement

Ethical review and approval was not required for the study on human participants in accordance with the local legislation and institutional requirements. Written informed consent for participation was not required for this study in accordance with the national legislation and the institutional requirements.

## Author Contributions

AL and JB both contributed to the conception and design of the work, collected data for the work, contributed to the analysis and interpretation of data for the work, drafted and revised the work, gave approval for the final version to be published, and agreed to be accountable for all aspects of the work in ensuring that questions related to the accuracy or integrity of any part of the work are appropriately investigated and resolved. During this work, we sought the collaboration with colleagues with a Muslim background, trained as physicians, others as anthropologists or as theological counselors, and followed the tenet of what they taught us when describing sociocultural attributions. Both authors contributed to the article and approved the submitted version.

## Conflict of Interest

The authors declare that the research was conducted in the absence of any commercial or financial relationships that could be construed as a potential conflict of interest.

## Publisher's Note

All claims expressed in this article are solely those of the authors and do not necessarily represent those of their affiliated organizations, or those of the publisher, the editors and the reviewers. Any product that may be evaluated in this article, or claim that may be made by its manufacturer, is not guaranteed or endorsed by the publisher.
