# Supplementary material for: Tactile and Somatic Hallucinations in a Muslim Population of Psychotic Patients
Source: Front Psychiatry. 2021 Oct 29;12:728397. doi: 10.3389/fpsyt.2021.728397 (PMC8586517; doi:10.3389/fpsyt.2021.728397)
Supplement: Supplementary file 1 [file Data_Sheet_1.PDF]

Hallucination Attribution List (HAL)

Translated from the Dutch by J.D. Blom, MD, PhD, Parnassia Psychiatric Institute, The Hague
© 2021

Instructions for use

The HAL aids to chart the phenomenological characteristics of hallucinations that are attributed to jinn (djinns, jnun). It is a semi-structured questionnaire, meaning that the order in which the various questions are asked is not essential. What is important though, is that all questions be answered. When for a given question more than one answer is applicable, all of them need to be scored.

Attention! This is a non-validated questionnaire developed for purely scientific purposes. It is not a diagnostic tool.

Date of interview:
Name of interviewer:
Patient number:

SECTION 1: HALLUCINATIONS GENERAL

Presence or absence of hallucinations, lifetime

1.0 Did you ever in your life perceive things that others could not perceive? For example, did you ever hear voices or have visions?
O Yes
O No → Stop interview

Level of consciousness

1.1 Did that ever happen while you were awake? Or was it always when you were asleep? Or during a transitional phase between sleep and wakefulness?
O While I was awake -> go to question 1.1.1
O Always during a transitional phase between sleep and wakefulness -> go to question 1.1.2.
O Always while asleep → Stop interview
O Unknown

Sensorium

1.1.1 Were you wide awake?
O Yes
O No, during such moments I was always sleepy/drowsy
O Unknown

Influence of substance use

1.2 Did you ever experience this without being under the influence of mind-altering substances such as alcohol, medicines or illicit substances?
O Yes
O No, I was under the influence of………. ……………………………………………………..
O Unknown

Open question, description of hallucinations

1.3 Would you mind telling me what you perceived?...............…………………….………
……………………………………………………………………………………………………
……………………………………………………………………………………………………
……………………………………………………………………………………………………
……………………………………………………………………………………………………
……………………………………………………………………………………………………
……………………………………………………………………………………………………
……………………………………………………………………………………………………

SECTION 2: RELIGIOSITY

Religious denomination, passive

2.1 Are you a Muslim?
O Yes
O No

Religious denomination, active

2.1.1 Are you a practising Muslim?
O Yes
O No

Reading the Quran

2.1.2 How often do you read the Qur'an?
O Every day
O At least once a week
O At least once per month
O At least once per year
O Rarely or never

Visiting the mosque

2.1.3 How often do you visit the mosque?
O Every day
O At least once a week
O At least once per month
O At least once per year
O Rarely or never

Evil eye, belief of others

2.2 Do you know anyone who believes in the evil eye?
O Yes, namely……………………………………………………………………………………
O No

Evil eye, own belief

2.2.1 Do you yourself believe in the evil eye?
O Yes
O No

Evil eye, own experience

2.2.2 Did you ever experience anything in which the evil eye played a role?
O Yes, namely……………………………………………………………………………………
O No
O I don't know

Magic, belief of others

2.3 Do you know anyone who believes in magic?
O Yes, namely……………………………………………………………………………………
O No

Magic, own belief

2.3.1 Do you yourself believe in magic?
O Yes………..…………………………………………………………………………………
O No
O I don't know

Magic, own experience

2.3.2 Did you ever experience anything in which magic played a role?
O Yes, namely……………………………………………………………………………………
O No
O I don't know

Jinn, belief of others

2.4 Do you know anyone who believes in jinn?

O Yes, namely…………………………………………………………………………………

O No

Jinn, own belief

2.4.1 Do you yourself believe in jinn?

O Yes

O No

O I don't know

Jinn, own experience

2.4.2 Did you ever experience anything in which jinn played a role?

O Yes, namely…………………………………………………………………………………

O No

O I don't know

SECTION 3: ATTRIBUTION

Attribution

3.1 Could it be that the visions/voices/experiences described by you before were caused by a jinn?

O Yes, it is possible

O No, that is impossible

O I don't know

O That is something I do not <u>want</u> to talk about -> <u>stop interview</u>

O That is something I am <u>not allowed</u> to talk about (because…………....) -> <u>stop interview</u>

Conviction

3.2 How sure are you that a jinn was at play?

O I am 100% certain that it was a jinn

O I think that it was a jinn, but I am not sure

O I am 100% certain that it was <u>not</u> a jinn

O I have no idea

Conviction, on Likert scale

3.2.1 Could you indicate on the scale below how certain you are that one or more jinn were involved?

0% (not)--100% (certain)

Alternative explanations

3.3 If your perceptual experiences were <u>not</u> caused by a jinn, what else could have been their cause?

O Allah

O God

O An angel (or angels)

O Iblis/Shaitan

O A spirit (or spirits) of deceased people, namely………………………..…………………….

O A living person, namely………….…………………………………………………………

O An animal (or animals), namely…………………………………………………………...

O A device, namely…………………………..…………………………………………………..

O My own brain or mind (due to illness or medicine use, for example)

O Magic

O The evil eye

O Something else, namely……………………………………………………………………

Open question, working mechanism of hallucinations

3.4 Can you explain <u>in which way</u> your visions/voices/experiences were caused by this?...
....
....
....
....
....
....
....

SECTION 4: PHENOMENOLOGICAL CHARACTERISTICS

Involvement with jinn

4.1 Were you ever involved with jinn?

O Yes
O No -> <u>stop interview</u>
O I don't know -> <u>stop interview</u>

Involvement with jinn, number

4.1.1 With how many jinn were you involved?

O One
O Several, namely...
O Several, but I have no idea how many

Classification of jinn

4.1.2 Were you involved with a

O Female jinn?
O Male jinn?
O Young jinn?
O Old jinn?
O Turkish jinn?
O Moroccan jinn?
O Jewish djinn?
O European jinn?
O Different, namely...

Visual hallucinations

4.2 Did you <u>see</u> the jinn with which you were involved?

O Yes
O No -> go to question 4.3

Jinn, visual aspects

4.2.1 What was the colour of the jinn you saw?

O No colour (including grey or black-and-white)
O Monochromatic, namely...
O Polychromatic, namely...
O Extremely bright or lively colours
O As if beaming and emanating a bright light
O Vague colours, as if bleached
O Vague colours, as if transparent
O Extremely dark or sombre colours
O Different, namely...

Jinn, size

4.2.2 How large was/were the jinn you saw?

O The size of an adult
O The size of a child
O Very small, namely...
O Very large, namely...
O Different, namely...

O I don’t know, because………………………………………………………………………………

Outward projection

4.2.3 Did you see the jinn outside yourself, the way you see me, for example?

O Yes
O No, I saw the jinn ‘in my mind’
O Different, namely..……………………………………………………………………………….
O I don’t know, because………………………………………………………………………………

Blending into background

4.2.3.1 Did you see the jinn occupying a natural place in your environment?

O Yes, the jinn stood/sat/lay on the ground/on a chair etc.
O No, the jinn appeared to float
O Different, namely…………………………………………………………………………………
O I don’t know, because………………………………………………………………………………

Background

4.2.3.2 Did you see the jinn against the background of your own surroundings?

O Yes
O No, the background was different from the place I was at
(namely……………………………………………………………………………………..…)
O Different, namely…………………………………………………………………………………
O I don’t know, because………………………………………………………………………………

Extracampine hallucinations

4.2.4 Did you see the jinn within the normal boundaries of your field of vision, or outside of it?

O Within the normal boundaries of my field of vision
O Outside my field of vision, namely above/below/next to me
O I saw the jinn with the back of my head
O I saw the jinn with a different body part, namely………………………………………...........
O Different, namely…………………………………………………………………………………
O I don’t know, because………………………………………………………………………………

Movement

4.2.5 Did the jinn show any movement?

O Yes, the jinn moved
O No, there was no movement
O I don’t know, because………………………………………………………………………………

Likeness

4.2.6 Did the jinn look like a familiar person, animal or character?

O Yes, it looked like an actual human being, namely.…………………………………………
O Yes, it looked like an animal, namely……………………………………………………….
O Yes, it looked like a familiar figure from an image/photograph/film, namely………………
O Yes, it looked like myself
O Different, namely…………………………………………………………………………………
O No
O I don’t know, because………………………………………………………………………………

Auditory hallucinations

4.3 Were you able to hear the jinn?

O Yes
O No -> <u>go to question 4.4</u>
O I don’t know, because………………………………………………………………………………

Jinn, auditory aspect, localisation

4.3.1 Did you hear the jinn inside or outside your head?

O Outside my head
O Inside my head
O I don’t know, because………………………………………………………………………………

Verbal auditory hallucinations

4.3.2 Did the jinn speak?

O Yes
O No -> go to question 4.3.6
O I don't know, because……………………………………………………………………………

Verbal auditory hallucinations, phenomenological aspects

4.3.2.1 What type of voice did the jinn have?

O A familiar male voice, namely………..……………………………………………………
O An unfamiliar male voice
O A familiar female voice, namely……..……………………………………………………
O An unfamiliar female voice
O A different type of voice, namely (a child's voice, an animal voice, a mechanical voice, etc.)……………………………………………………………………………………….............
O My own voice
O I don't know, because……………………………………………………………………………

Verbal auditory hallucinations, interaction

4.3.2.2 Were there any voices conversing with each other or talking simultaneously?

O Yes, namely……………………………………………………………………………………
O No
O I don't know, because……………………………………………………………………………

Intelligibility

4.3.2.3 Could you properly hear what the jinn were saying?

O Yes -> go to question 4.3.3
O No

Reasons for unintelligibility

4.3.2.3.1 Why were you unable to properly hear what the jinn were saying?

O The voice was too soft
O The voice sounded as if from far away
O Voices were speaking simultaneously
O They spoke a language I do not understand, namely………………………………………..
O There were too many other sounds, namely…………………………………………………
O Different, namely..……………………………………………………………………………..

Content, open question

4.3.3. Would you mind telling me what they said?………………………………………….
……………………………………………………………………………………………………….
.……………………………………………………………………………………………………
……………………………………………………………………………………………………….

Content, origin

4.3.3.1 What did this content make you think of?

O They were things I had *never* heard before
O Things that are in the Qur'an or which I heard in the mosque
O Things that other people, outside the mosque, once said to me
O Things that I heard in a film or on the radio
O Things that I myself once said
O Different, namely………………………………………………………………………………

Content, repetitive nature

4.3.4 Were there any words (or sentences or phrases) that were repeated over and over?
(-> Interviewer gives as an example, "I'll be there, I'll be there, I'll be there")

O Yes, sometimes it is like listening to a broken record
O No
O Different, namely………………………………………………………………………………

Relation with the interviewee

4.3.5 Do the voices interfere with you?

O Yes, they provide me with useful information
O Yes they provide critical comments
O Yes, they give commands
O No
O Different, namely……………………………………………………………………………

Musical aspect

4.3.6 Do you ever hear any music while being in contact with a jinn?

O Yes, with vocals
O Yes, instrumental
O No

Nonverbal auditory hallucinations

4.3.7 Do you ever hear any other sounds while being in contact with a jinn?

O Yes
O No -> go to question 4.4

Nonverbal auditory hallucinations, aspect

4.3.7.1 What other sounds do you hear?

O Humming
O White noise
O Crackling
O Squeaking
O Ticking
O Knocking
O The doorbell
O The telephone
O Other electrical devices
O Traffic
O Different, namely……………………………………………………………………………

Olfactory hallucinations

4.4 Do you ever smell any strange odours while being in contact with a jinn?

O Yes
O No -> go to question 4.5

Olfactory hallucinations, aspect

4.4.1 What odours did you smell?

O Gas
O Sulphur
O Feces
O Perfume
O Flowers
O Food, namely……………………………………………………………………………..
O Different, namely……………………………………………………………………………

Gustatory hallucinations

4.5 Do you ever experience a strange taste while being in contact with a jinn?

O Yes
O No -> go to question 4.6

Gustatory hallucinations, aspect

4.5.1 What did you taste?

O Garlic
O Other food, namely……..……………………………………………………………………..
O Feces
O Different, namely……………………………………………………………………………

Tactile hallucinations

4.6 Were you ever physically touched by a jinn?

O Yes
O No -> go to Section 5

Tactile hallucinations, aspect

4.6.1 What did you feel when you were being touched?

O As if I were being pushed
O As if I were hit
O As if I were stabbed
O The wind
O Water
O Electricity
O As if a jinn entered my body
O As if I had sexual contact
O Different, namely…………………………………………………………………………………

Tactile hallucinations, other

4.6.2 Did you ever feel anything else on your skin or inside your body while being in contact with a jinn?

O Yes, namely……………………………………………………………………………………
O No

SECTION 5: HALLUCINATIONS, LIFETIME

Debut of hallucinations

5.0. Can you recall when for the first time in your life you came into contact with a jinn?

O Yes, it was the year ….
O The jinn has/have always been there
O No

Debut of hallucinations, activity

5.1. Can you recall what you were doing at the time?

O I slept
O I was somewhere between waking and sleeping
O I was awake and I was (talking, walking, praying, etc.)……………………………………...
…………………………………………………………………………………………………..
…………………………………………………………………………………………………..
O I was ill, I suffered from (name of affliction/description of complaints)..……………………
O I had just taken medication, namely……………………………………………………………
O I had just used alcohol, namely………………..…………………………..………………......
O I had just used an illicit substance, namely……………………………………………………
O I had been under great mental pressure, namely…………………………………………….
O I had done something wrong, namely………………………………………………………….
O I was under someone's spell, namely………………………………………………………….
O Different, namely…………………………………………………………………………………
O No

Debut of hallucinations, first reaction

5.2 What was your immediate reaction when you first came into contact with a jinn?

O I don't recall
O I started
O I got afraid
O I was surprised, but not afraid
O I became suspicious
O I tried to act as if nothing were the matter
O I was not surprised, I had been expecting something like this for a long time
O I was happy
O Different, namely…………………………………………………………………………………

Frequency

5.3 How often, on average, are you involved with (a) jinn?

O Just once
O A few times
O Not every day
O Every day
O Several times a day
O Several times per hour
O Continuous, all day long
O Different, namely…………………………………………………………………………………

SECTION 6: FINAL QUESTIONS

Influence on jinn

6.0 Do you have any influence on the jinn?

O Yes, I can (sometimes) make them go away
O Yes, I can (sometimes) make them do something different than what they want
O Yes, I can (sometimes) summon them
O No, they come and go at their own time → Go to question **6.2**
O No, they are always there → Go to question **6.2**
O Different, namely…………………………………………………………………………………

Influence on jinn, mechanism of action

6.1 In which way do you influence the jinn?

O By praying
O By reading the Qur'an
O By distracting myself, namely……………………………………………………………………..
O By doing what they tell me to do, namely…………………………………………………..
O By silently subjecting them to my will
O By negotiating with them
O By speaking to them out loud
O By taking medication, namely………..…………………………………………………………..
O By using a different substance, namely……………………………………………………….
O Different, namely…………………………………………………………………………………

Own wish regarding the jinn

6.2 Would you like to be rid of the jinn?

O Yes
O No, because………………………………………………………………………………………
O I don't know

Therapeutic options

6.3 Which of the following methods do you think might help to get rid of a jinn?

O Prayer
O Reading the Qur'an
O Visiting the mosque
O Herbal treatment
O Dissolving Qur'anic texts in water and drinking them
O Pilgrimage
O By restoring a previously made mistake
O Medications (antipsychotics)
O Different, namely…………………………………………………………………………………
O I don't know

Subjective typification of jinn

6.4 Would you mind indicating which of the following statements comply best with the way you experience jinn?

O "Those jinn are real, I am surprised that you don't see or hear them."
O "Those jinn are proof that there is more between Heaven and earth."
O "Whether I will ever be rid of those jinn is in Allah's hands."

O “That I am involved with those jinn is because I have an illness.”
O “Those jinn are merely my own thoughts.”
O “Those jinn are like dreaming during the day.”
O “Those jinn ruined my life.”
O “Those jinn enriched my life.”
O Different, namely…………………………………………………………………………….

Other

6.5 Are there any other issues, pertaining to the jinn that you experienced, which you would like to mention in the context of the present study?
